# Endoscopic endonasal transsphenoidal surgery for unusual sellar lesions: eight cases and review of the literature

**DOI:** 10.3389/fneur.2024.1309691

**Published:** 2024-02-13

**Authors:** Jiandong Wu, Peng Deng, Jinhong Qian, Yanli Lu, Zhiliang Ding, Xiaolong Hu, Yuhui Gong, Xiaoyu Tang, Mian Ma

**Affiliations:** ^1^Department of Neurosurgery, The Affiliated Suzhou Hospital of Nanjing Medical University, Suzhou, Jiangsu, China; ^2^Department of Radiology, The Affiliated Suzhou Hospital of Nanjing Medical University, Suzhou, Jiangsu, China

**Keywords:** endoscopic endonasal surgery, sellar lesions, solitary fibrous tumor, lymphocytic hypophysitis, cavernous sinus hemangiomas, ossifying fibroma, Mucocele, pituitary abscess

## Abstract

**Background:**

Preoperative imaging for some unusual lesions in the sellar region can pose challenges in establishing a definitive diagnosis, impacting treatment strategies.

**Methods:**

This study is a retrospective analysis of eight cases involving unusual sellar region lesions, all treated with endoscopic endonasal transsphenoidal surgery (EETS). We present the clinical, endocrine, and radiological characteristics, along with the outcomes of these cases.

**Results:**

Among the eight cases, the lesions were identified as follows: Solitary fibrous tumor (SFT) in one case, Lymphocytic hypophysitis (LYH) in one case, Cavernous sinus hemangiomas (CSH) in one case, Ossifying fibroma (OF) in two cases; Sphenoid sinus mucocele (SSM) in one case, Pituitary abscess (PA) in two cases. All patients underwent successful EETS, and their diagnoses were confirmed through pathological examination. Postoperatively, all patients had uneventful recoveries without occurrences of diabetes insipidus or visual impairment.

**Conclusion:**

Our study retrospectively analyzed eight unusual lesions of the sellar region. Some lesions exhibit specific imaging characteristics and clinical details that can aid in preoperative diagnosis and inform treatment strategies for these unusual sellar diseases.

## Introduction

The most commonly detected masses in the sellar region are predominantly pituitary adenomas, accounting for about 90%. Some unusual lesions, such as craniopharyngiomas, meningiomas, Rathke’s cysts, or chordomas, represent nearly 10% (1, 2). While some lesions often present with characteristic radiological features suggesting a preoperative diagnosis, clinicians sometimes encounter unusual sellar lesions that lack typical imaging findings, potentially leading to preoperative imaging misdiagnoses (3, 4). In our case series, we present a group of unusual sellar diseases treated via endoscopic endonasal transsphenoidal surgery (EETS) (5). We aim to describe their clinical, endocrine, and radiological characteristics and outcomes, ultimately assisting in preoperative diagnosis and providing rational treatment strategies.

## Methods

### Clinical data

This study is a retrospective analysis of eight cases of unusual sellar region lesions treated with the EETS approach at Suzhou Municipal Hospital from October 2020 to March 2023. The cohort includes three females and five males, aged 18 to 73 years. Three cases of sellar lesions preoperative MRI were misdiagnosed as pituitary adenoma, including Solitary fibrous tumor (SFT) in one case, Lymphocytic hypophysitis (LYH) in one case, and Cavernous sinus hemangiomas (CSH) in one case. Three lesions with typical imaging manifestations, but located unusually, include Sphenoidalia ossifying fibroma (OF) in two cases and Sphenoid sinus mucocele (SSM) in one case. Two cases of Pituitary abscess (PA) preoperative MRI were misdiagnosed as Rathke’s cyst. All patients received EETS from the same surgical team. Post-surgery, all cases underwent pathological diagnosis and immunohistochemistry evaluation. Clinical and imaging information of the patients was also collected and analyzed (Tables 1, 2).

**Table 1:** Clinical information of the eight cases.

Cases no.	diagnose	Sex	age	headache	visual disturbance	Eye discomfort	Endocrine abnormality	other
1	SFT	female	43	yes	no	Yes	normal	–
2	LYH	female	27	yes	left eye: Blind right eye: Visual field defect		Low ACTH	Pregnant
3	CSH	male	61	no	no	yes	normal	mild diplopia
4	OF	male	18	no	no	no	normal	Nasal discomfort
5	OF	male	33	yes	no	yes	norma	Nasal discomfort
6	SSM	male	63	yes	no	yes	normal	–
7	PA	female	67	yes	no	yes	normal	Intermittent fever
8	PA	male	72	yes	no	no	normal	History of sinusitis;

SFT, Solitary fibrous tumor; LYH, Lymphocytic hypophysitis; CSH, Cavernous sinus hemangiomas; OF, Ossifying fibroma; SSM, Sphenoid sinus mucocele; PA, Pituitary abscess.

**Table 2:** Imaging information of the eight cases.

Cases no. & diagnose	size (mm)	solid	Cystic component	MRI			key point
				T1WI	T2WI	TIWI Enhancement	
1. SFT	30*20 mm	yes	no	isodense	isodense	homogeneous hyperintense	The enhanced signal was higher than pituitary tissue
2.LYH	30*22 mm	yes	yes	inhomogeneous isodense	inhomogeneous isointense	hyperintense	The lesion grew symmetrically and could not be distinguished from the pituitary tissue
3.CSH	30*25 mm	yes	no	hypointense	inhomogeneous hyperintense	hypointense	The tumor did not significant enhancement, the margin was enhanced.
4.OF	40*25 mm	yes	no	hyperintense	hyperintense	–	The single butterfly bone is damaged; T1WI: hyperintense
5.OF	45*30 mm	yes	yes	hypointense	inhomogeneous hyperintense	hyperintense	Left sphenoid bone hyperplasia with ground glass shadow; T2WI: cystic component
6.SSM	32*28 mm	no	yes	hyperintense	hypointense	hyperintense	unusual location
7.PA	15*15 mm	no	yes	hypointense	inhomogeneous hyperintense	hypointense	DWI: local hyperisignal; The pituitary gland and pituitary stalk were enhanced uniformly
8.PA	15*15 mm	no	yes	hypointense	inhomogeneous hyperintense	hypointense	Nasosinusitis; DWI: local hyperisignal; The pituitary gland and pituitary stalk were enhanced uniformly

SFT, Solitary fibrous tumor; LYH, Lymphocytic hypophysitis; CSH, Cavernous sinus hemangiomas; OF, Ossifying fibroma; SSM, Sphenoid sinus mucocele; PA, Pituitary abscess.

## Results

### Patient characteristics

#### Solitary fibrous tumor (case 1)

**Figure 1:**
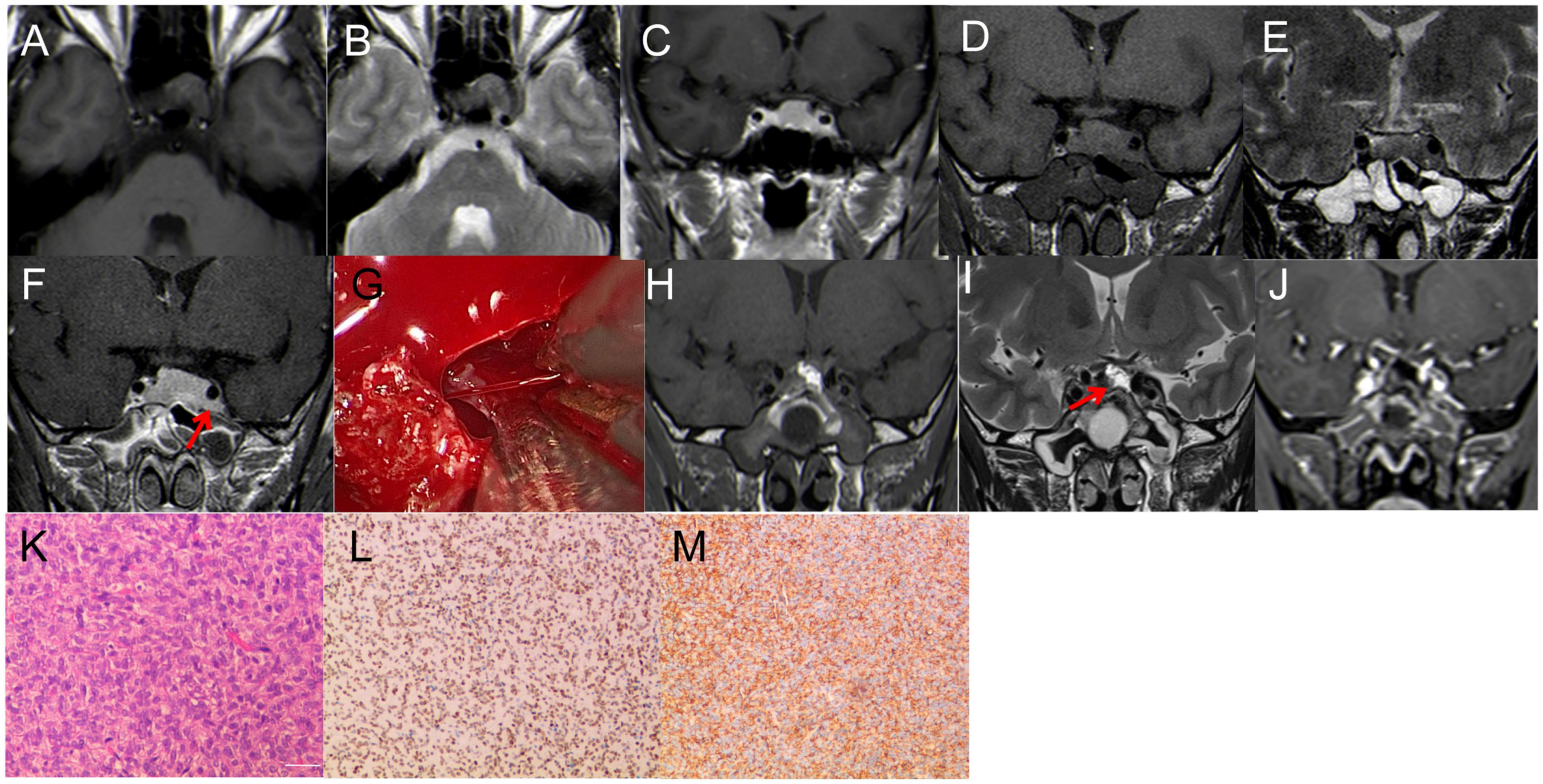
**(A–C)** First MRI. **(A)** T1WI: isodense signal, **(B)** T2-MRI: isodense signal. **(C)** Enhanced T1WI: Significant enhancement, size 20 mm*18 mm*15 mm. **(D–F)** Review 3 months after the first surgery. **(D)** T1WI: isodense signal; **(E)** T2WI: isodense signal; **(F)** enhanced T1WI: Significant enhancement, size 30 mm*20 mm*18 mm. **(G)** During the operation, it was found that the tumor invaded the left cavernous sinus, and the blood vessels supplying the tumor bleeding was obvious, and the hemostasis was effectively stopped by bipolar electrocoagulation. **(H–J)** Three days after the second surgery. **(H–I)** T1WI; I: T2WI,The high signal indicated by the red arrow is the fat tissue; **(J)** Enhanced T1WI, satisfactory tumor resection with no residual. **(K–L)** Pathological examination, **(K)** H&E staining (×200); **(L–M)** immunohistochemistry (×200); CD 99 (+), STAT6 (+).

A 43-year-old female experienced blurred vision in her left eye after undergoing surgery for a tumor in the sellar region 3 months prior. The initial MRI at another hospital revealed a solid tumor measuring 20 mm × 18 mm in the pituitary fossa, misdiagnosed as a pituitary tumor (Figures 1A–C). Subsequently, the patient underwent a partial resection of the tumor in the saddle area using a microscopic transsphenoidal approach due to its rich blood supply. Pathological diagnosis confirmed the tumor as SFT with a Ki-67:10%. Three months later, a follow-up MRI at our hospital showed significant enlargement of the tumor to 30 mm × 20 mm, with invasion into the bilateral cavernous sinuses (Figures 1D–F). The patient then underwent EETS. Intraoperatively, the tumor was identified as having a firm, tan-white consistency, distinct from pituitary adenomas, and had invaded the left cavernous sinus. Notably, significant bleeding occurred during tumor removal; hemostasis was effectively achieved by bipolar electrocoagulation under direct vision of the neuroendoscope (Figure 1G). Postoperative MRI showed satisfactory tumor clearance (Figures 1H–J). The patient experienced transient diabetes insipidus postoperatively but had no pituitary dysfunction or visual impairment. Histology and immunohistochemical staining confirmed the diagnosis of SFT (Figures 1K–M). Post-surgery, she received proton beam therapy to minimize tumor recurrence. At one-year follow-up, the patient’s condition was stable.

### Lymphocytic hypophysitis (case 2)

**Figure 2:**
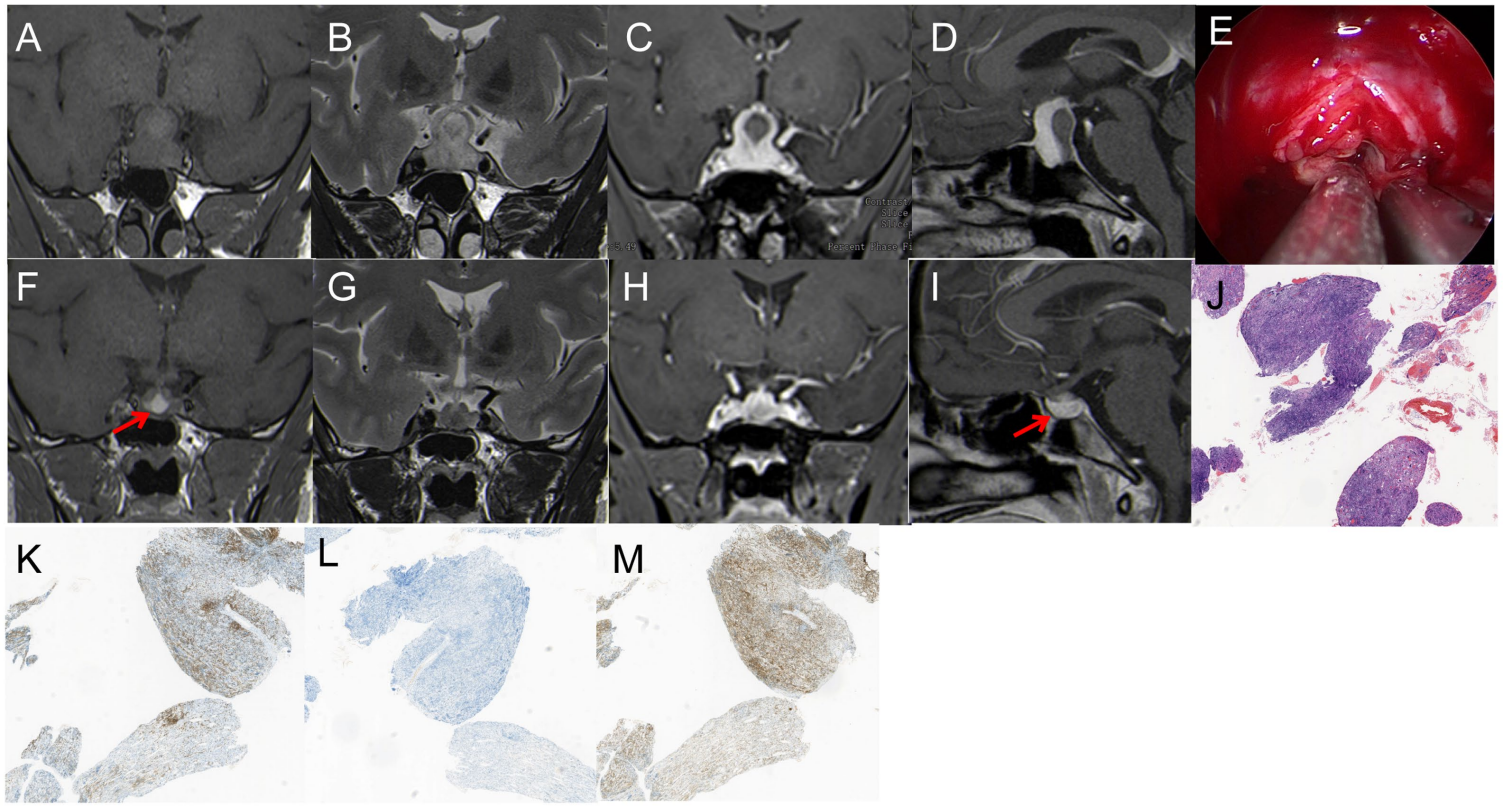
**(A,B)** T1WI and T2WI showed iso-hyper signal; **(C,D)** enhancement on T1WI and no enhancement in the cystic area, showing the “snowman” sign, 30 mm*22 mm in size. **(E)** The surrounding tumor tissue presented with pronounced fibrosis and a firm texture, markedly different from a typical pituitary tumor. **(F–I)** MRI showed that the tumor had disappeared after two months; **(F)** T1WI showed inhomogeneous hyperintense signal, red arrow showed cystic hypersignal; **(G)** T2WI: inhomogeneous hypointense signal. **(H,I)** Enhanced T1WI: Significant enhancement, red arrow showed pituitary. **(K–M)** Pathological examination, **(J)** H&E staining (×40) The disorder and destruction of pituitary tissue caused by lymphoplasmic cell inflammation and fibrosis; **(K–M)** immunohistochemistry (×40): **(K)** CD 20 (+), **(L)** CD 38 (+), **(M)** CD 43 (+).

A 27-year-old woman, 37+ weeks pregnant, presented with a one-month history of headache and bilaterally diminished vision, nearing blindness in the left eye. Prenatal tests indicated mild hypothyroidism. Throughout her pregnancy, her cortisol levels were normal, but her thyroid function was slightly reduced (Total triiodothyronine: 0.73 nmol/L; Free triiodothyronine: 2.3 pmol/L). A reevaluation of hormone levels 3 days post-delivery showed a substantial drop in cortisol (27.6 nmol/L), while other hormone levels were normal. She had no personal or family history of autoimmune diseases. MRI revealed a mass lesion with cystic changes in the sellar region, breaking through the diaphragm of the saddle and showing the “snowman” sign. T1WI and T2WI showed iso-hyper signal, with significant enhancement on T1WI and no enhancement in the cystic area. The lesion grew symmetrically and was indistinguishable from the pituitary tissue, measuring 30 mm × 22 mm (Figures 2A–D). Treatment included EETS and hormonotherapy. During surgery, cystic fluid resembling that of a Rathke’s cyst was released. However, the surrounding tumor tissue had pronounced fibrosis and a firm texture, markedly different from a typical pituitary tumor (Figure 2E). Immediate pathology indicated pituitary inflammation, but the specific subtype was unidentified. To prevent permanent hypopituitarism after full mass removal, only a small sample of the lesion was excised for routine pathology. Histopathological examination confirmed the lesion as LYH. Immunohistochemistry showed positivity for epithelial cells: CgA, Syn, CK18, CKpan, EMA, CD2-40; lymphocytes: CD3, CD5, CD20, CD38, CD43, CD79α, CD138, Ki67 (5–15%; Figures 2J–M). Post-surgery, the patient exhibited mental apathy, reduced appetite, and slight improvement in vision, without diabetes insipidus symptoms. Blood hormone tests indicated low cortisol. Hormonotherapy was administered: methylprednisolone sodium succinate 60 mg/d × 3d, followed by a reduction to 40 mg/d × 3d, then oral prednisolone tablets 30 mg/d, with a weekly decline of 5 mg, then maintained at 5 mg/d. Three-month follow-up MRI showed lesion disappearance (Figures 2F–I), and the patient’s vision returned to normal.

### Cavernous sinus hemangiomas (case 3)

**Figure 3:**
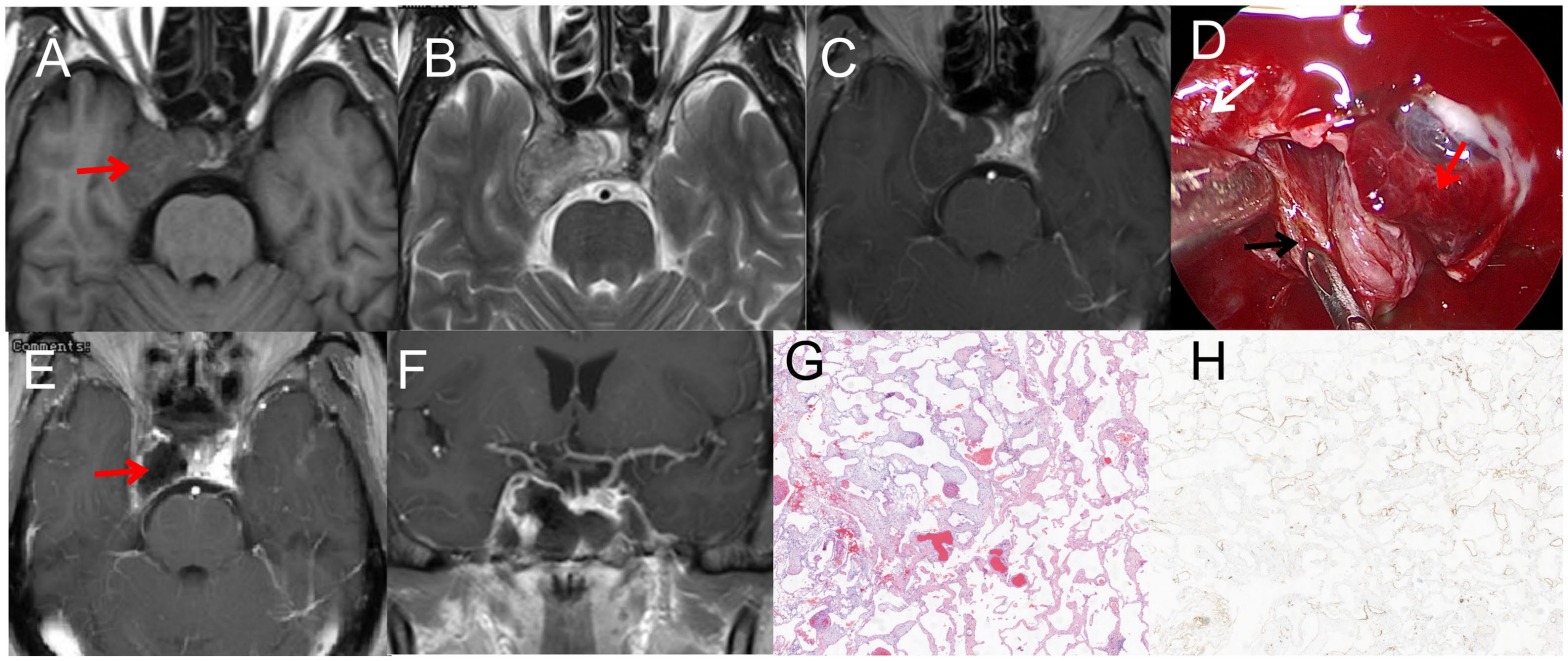
**(A)** T1WI iso-signal, the red arrow indicated that the tumor is located in the right cavernous sinus **(B)** T2WI iso-hyper mixed signal. **(C)** T1WI enhancement: The tumor did not significant enhancement, the margin was enhanced, 30mm*30mm in size; **(D)** Normal pituitary and tumor tissue were fully exposed during the operation, the red arrow indicated the pituitary, the black arrow indicated the tumor, the white arrow indicated the internal carotid artery. **(E–F)** Postoperative MRI, the red arrow indicated satisfactory resection of the tumor. **(G)**: H&E staining (×40); **(H)** immunohistochemistry (×40), CD 34(+).

A 61-year-old man presented with a two-week history of headache, numbness in the right frontal area, and mild diplopia. Visual field perimetry showed normal results. MRI revealed a mass lesion in the right parasellar region, with T1 iso-signal and T2 iso-hyper mixed signal. The tumor showed no significant enhancement, but the margin was enhanced. It measured 30 mm × 30 mm (Figures 3A–C). The patient underwent EETS for treatment. The dura was incised using a sickle knife, starting from the dural membrane at the base of the saddle. No tumor was found surrounding the pituitary gland. The incision in the dural membrane was extended laterally toward the right internal carotid artery (ICA), where reddish tumor tissue was identified. After removing the tumor’s enveloping membrane, the tumor noticeably contracted and extended into the medial cavernous sinus (Figure 3D). Hemostasis, due to the tumor’s abundant blood supply, was effectively achieved using cotton tablets and Surgiflo. Under direct visualization, the tumor was carefully mobilized from the cavernous sinus. The base of the saddle was repaired to prevent cerebrospinal fluid leakage (Figures 3E,F). As a result, the patient’s symptoms disappeared, and eye movements and hormone levels normalized. Pathology examination revealed CSH (Figures 3G,H). After 1 year of follow-up, the patient’s condition was stable.

### Ossifying fibroma (case 4)

**Figure 4:**
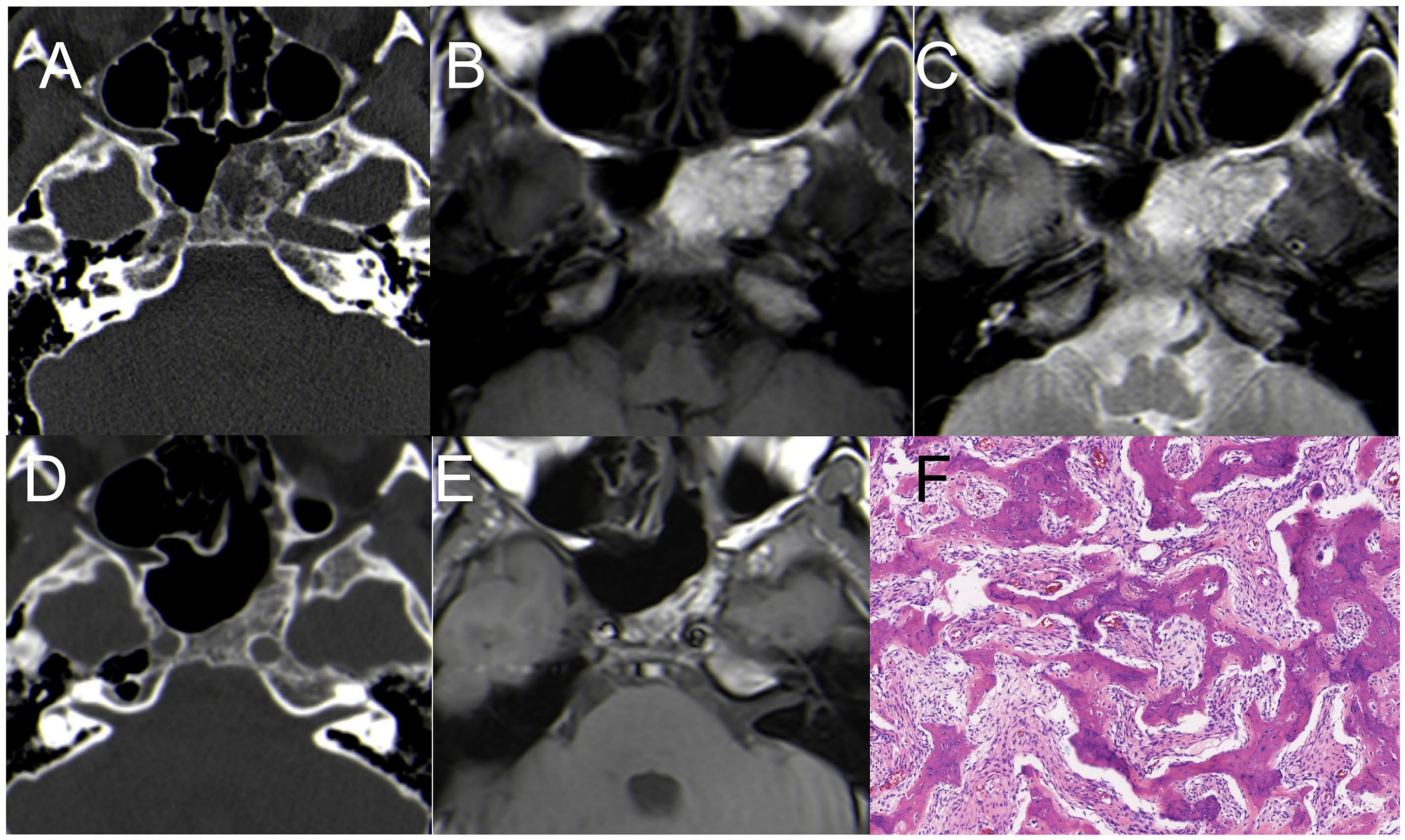
Case 4 CT + MRI. **(A)** CT scan showed uneven density in the left sphenoid bone **(B)** hyper-signal on T1WI; **(C)** hyper-signal on T2WI; **(D)** Postoperative CT indicated satisfactory resection of the tumor. **(E)** Postoperative MRI (T1WI) indicated satisfactory resection of the tumor. **(F)** H&E staining demonstrating features typical of OF (×200).

An 18-year-old man presented with nasal discomfort and a two-month history of headache. CT scan showed uneven density in the left sphenoid bone, a central swelling growth in the bone marrow cavity, an incomplete bony envelope at the edge, and a dense bone-like septum in the tumor. MRI revealed a lesion measuring 45 mm × 25 mm, with hyper-signal on T1WI and T2WI (Figures 4A–C). The patient underwent EETS; the bone shell on the skull base was gradually peeled off, utilizing a diamond drill toward the tumor shell until the bone cortex was smoothened. Pathology examination revealed OF (Figure 4F). Six months postoperative follow-up CT and MRI confirmed complete removal of the tumor (Figures 4D,E).

### Ossifying fibroma (case 5)

**Figure 5:**
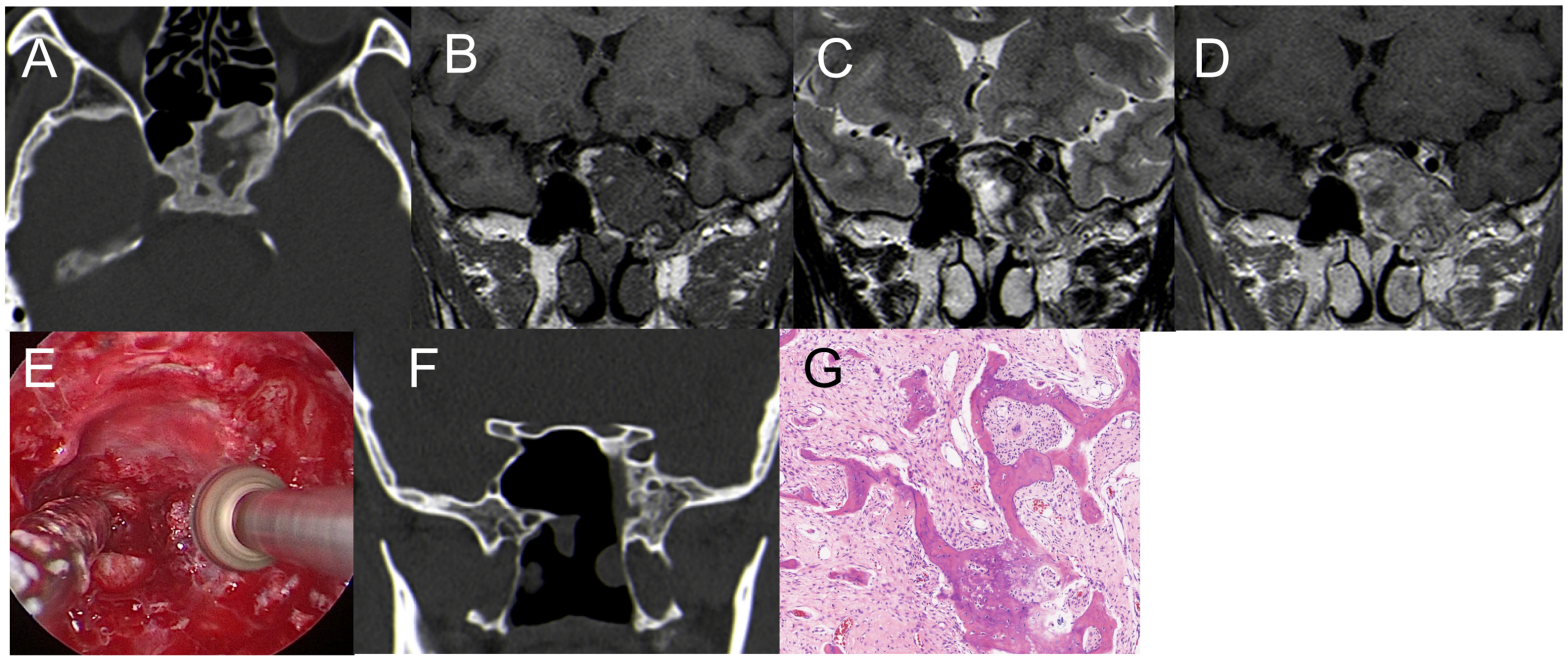
Case 5 CT+ MRI **(A)** CT scan showed uneven density in the left sphenoid bone **(B)** hypo-signal on T1WI; **(C)** inhomogeneous hyperintense signal on T2WI; **(D)** T1WI enhancement: The tumor significant enhancement, 45 mm*30 mm in size. **(E)** The calcified lesions were removed with a drill. **(F)** Postoperative CT indicated small residual tumor in the left sphenoid bone. **(G)** H&E staining demonstrating features typical of OF (×200).

A 33-year-old man presented with nasal discomfort and headache for 1 years. CT scan showed uneven density in the left sphenoid bone, with a central swelling growth in the bone marrow cavity, an incomplete bony envelope at the edge. MRI revealed a cystic lesion measuring 45 mm × 30 mm, with hypo-signal on T1WI and inhomogeneous hyperintense on T2WI, with significant enhancement on T1WI (Figures 5A–D). The patient underwent EETS, after removing the anterior wall of the sphenoid sinus, tumor tissue becomes visible, characterized by abundant blood supply, following the partial excision of cystic lesions, utilizing a diamond drill toward the tumor shell until the bone cortex was smoothened (Figure 5E). Pathology examination revealed OF (Figure 5F). Six months postoperative follow-up CT and MRI confirmed complete removal of the tumor (Figure 5E).

### Sphenoid sinus mucocele (case 6)

**Figure 6:**
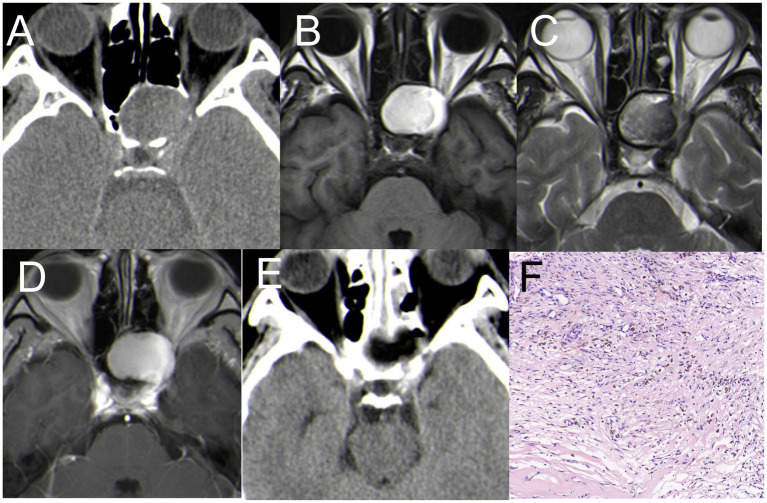
**(A)** CT: a lesion in the sphenoid sinus, with obvious dilation and bone destruction on the left side **(B)** hyper-signal on T1WI; **(C)** hypo-signal on T2WI; **(D,E)** Coronal enhancement of T1WI showed obvious enhancement, size 35*30 mm. **(F)** Postoperative CT indicated satisfactory resection of the tumor. **(F)** H&E staining demonstrating features typical of mucocele (×200).

A 63-year-old male presented with headache, left eye soreness, and discomfort for 1 month. CT scan showed a lesion in the sphenoid sinus, with obvious dilation and bone destruction on the left side. The lesion measured 35 mm × 30 mm, compressing the left orbital wall. MRI showed hyper-signal on T1WI, hypo-signal on T2WI, and enhanced signal on T1WI after enhancement (Figures 6A–D). The patient underwent EETS and sphenoid sinus opening surgery. Opening the sphenoid sinus wall revealed a viscous yellowish liquid mucocele. The mucocele was removed under the direct vision of neuroendoscopy, and the sphenoid sinus opening was maintained. This method keeps the cavity open, preserving the continuity of the sinus mucosa with the nasal cavities, thereby reducing the risk of recurrence. Postoperative pathological diagnosis confirmed mucocele. Pathology examination revealed sphenoid sinus mucocele with cystic wall fibrosis and inflammatory cell response (Figure 6F). After surgery, the patient’s symptoms resolved. At six-month follow-up, the patient’s condition remained stable.

### Pituitary abscess (case 7)

**Figure 7:**
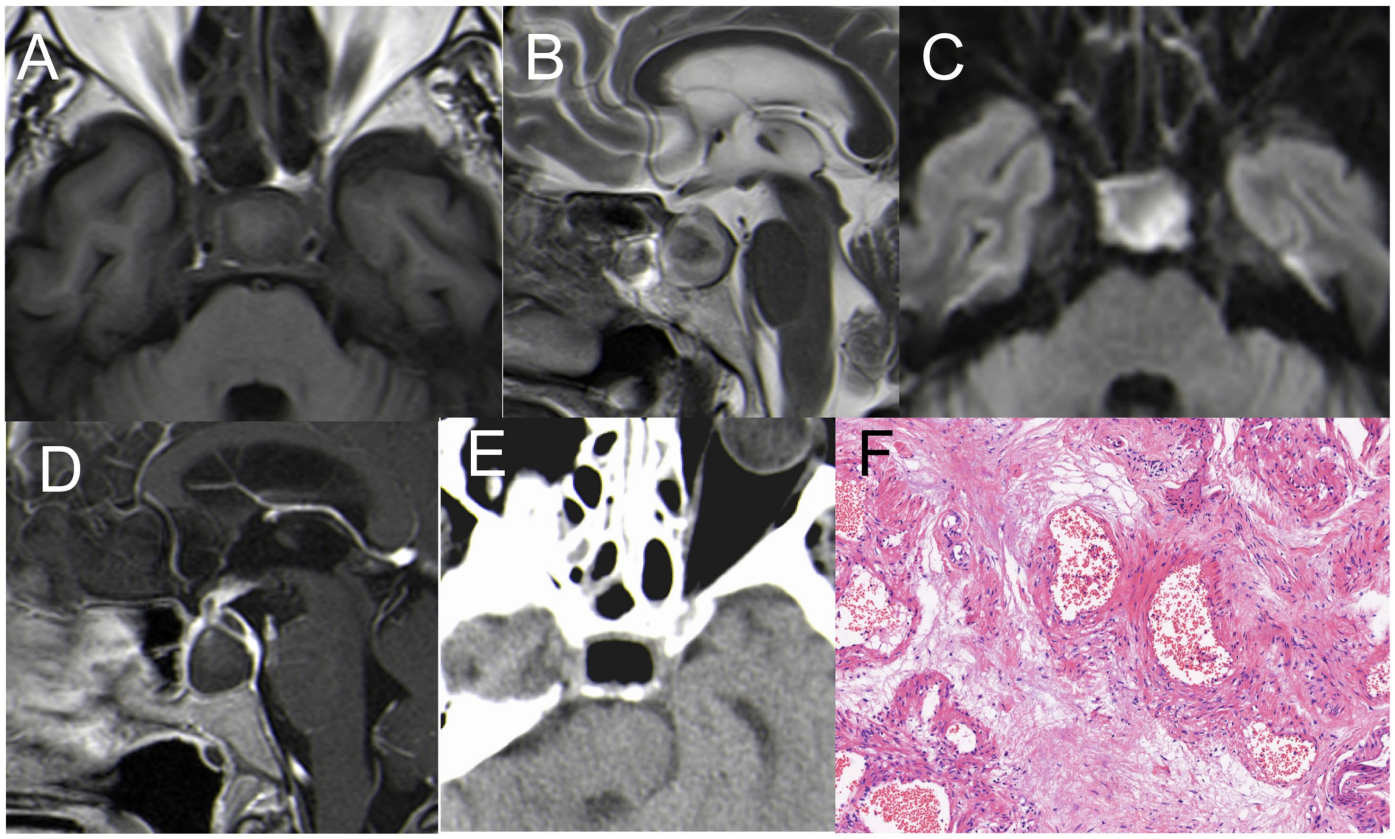
**(A)** Hypointense on T1WI; **(B)** inhomogeneous hyperintense on T2WI; **(C)** hyperintense on DWI; **(D)** The pituitary gland and pituitary stalk were significantly strengthened. **(E)** Postoperative CT indicated satisfactory resection of the PA. **(F)** H&E staining (×200): A large number of inflammatory cells infiltrated with abnormal hyperplasia of blood vessels.

A 67-year-old woman presented with a one-month history of headache and intermittent fever. Physical examination and visual field perimetry were normal, and hormone levels were also normal. CT scan showed abnormal density in the pituitary fossa, and MRI revealed a cystic lesion in the sellar region measuring 15 mm × 15 mm. The lesion showed mixed iso-signal on T1WI, mixed hyperintense on T2WI, and mixed hyperintense on DWI. The pituitary gland and stalk were significantly enhanced (Figures 7A–D). Initially, the patient received medical therapy with Vancocin and ceftriaxone sodium (Vancocin 0.5 g ivgtt q8h, ceftriaxone sodium ivgtt 2.0 qd), but symptoms persisted after 3 weeks. Subsequently, the patient underwent EETS, when the bottom of the saddle was opened, yellow-white pus flowed from the bottom of the saddle. The pituitary fossa was repeatedly rinsed with hydrogen peroxide during the operation, and the bottom of the saddle was not repaired. Postoperative CT indicated satisfactory resection of the PA (Figure 7E), Pathological smears showed abundant neutrophils and degenerated pituitary cells (Figure 7F). Post-surgery, symptoms resolved, hormone levels normalized, no cerebrospinal fluid rhinorrhea and no visual defects were observed.

### Pituitary abscess (case 8)

**Figure 8:**
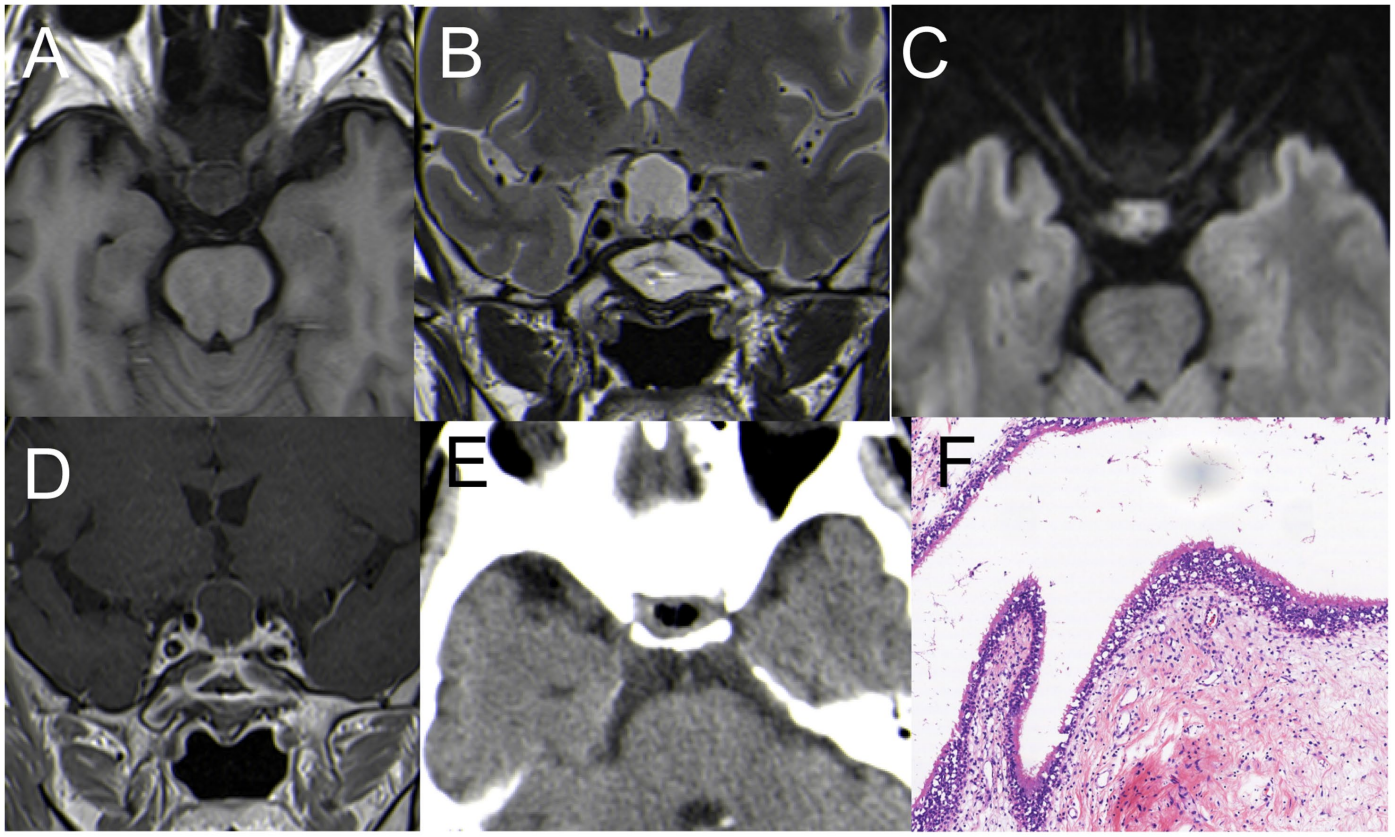
**(A)** Hypointense on T1WI; **(B)** inhomogeneous hyperintense on T2WI; **(C)** hyperintense on DWI. **(D)** The pituitary gland and pituitary stalk were significantly strengthened. **(E)** Postoperative CT indicated satisfactory resection of the PA. **(F)** H&E staining (×200): A large number of inflammatory cells infiltrated with abnormal hyperplasia of blood vessels.

A 72-year-old male patient complained of headaches and eye strain but did not present with fever. MRI revealed a cystic lesion in the sellar region measuring 15mm×15mm. The lesion showed hypo -signal on T1WI, hyperintense on T2WI, and mixed hyperintense on DWI. The Cyst margin were significantly enhanced (Figures 8A–D). Initially misdiagnosed as a cyst pre-surgery, the patient later underwent EETS. During the procedure, the mucosa of the sphenoid sinus was thickened, resembling sphenoid sinusitis. After clearing the inflamed sphenoid sinus mucosa, an abscess was observed flowing from the sellar bottom. The internal abscess was connected with the sphenoid sinus mucosal abscess due to destruction of the sellar bottom bone. With visible abscess discharge in the pituitary fossa, the diagnosis was revised to pituitary abscess. Postoperative CT indicated satisfactory resection of the PA (Figure 8E), Pathological smears showed a large number of inflammatory cells infiltrated. (Figure 8F). Post-surgery, symptoms resolved, hormone levels normalized, no cerebrospinal fluid rhinorrhea and no visual defects were observed.

## Discussion

Sellar tumors constitute 10–15% of all intracranial tumors, with pituitary adenomas representing 90% of these cases (6). Our study presents eight cases of unusual sellar lesions encountered in our neurosurgery department from October 2020 to March 2023. These cases were treated via EETS, a technique that has become increasingly preferred for sellar lesions surgery with minimal postoperative complications over the past 20 years (7–10). All diagnoses were confirmed through pathological examination, though some presented challenges in making a definitive diagnosis based solely on imaging examinations or clinical manifestations. We analyzed the causes of preoperative imaging misdiagnoses in this group and aimed to improve the preoperative diagnosis rate and provide a reasonable treatment strategy.

1. Three cases of sellar lesions preoperative MRI were misdiagnosed as pituitary adenoma

Three patients were misdiagnosed with pituitary adenoma based on preoperative MRI. These included Case 1 (SFT), Case 2 (LYH), and Case 3 (CSH).

(1) Solitary Fibrous Tumors (SFTs) are rare fibroblastic tumors of mesenchymal origin, accounting for about 1% of all primary CNS tumors (11). Their occurrence in the saddle area is exceedingly rare, with only a few case reports documented (12). Consequently, SFTs are easily misdiagnosed as pituitary adenomas. SFTs are usually solitary with lobulated growth and irregular morphologies. They often attach to adjacent meninges with a narrow base and exhibit invasive characteristics (12–14). In Case 1, an MRI performed at an outside hospital showed a 20 mm × 18 mm solid tumor in the pituitary fossa. Preoperative imaging findings were indistinguishable from those of pituitary adenoma, leading to its initial misdiagnosis. The patient first underwent a microscopic transsphenoidal resection of the tumor in the saddle area. However, due to its abundant blood supply and bleeding from the cavernous sinus, only a portion of the tumor was removed. Postoperative pathology confirmed the tumor as SFT. A follow-up MRI 3 months later at our hospital displayed significant tumor growth, measuring 30 mm × 20 mm, with invasion into the bilateral cavernous sinuses (Figures 1D–F). A detailed examination of the MRI revealed a rightward shift of the pituitary stalk, with the pituitary tissue positioned to the right. The tumor displayed pronounced enhancement, contrasting with the subdued enhancement signal of the pituitary, suggesting a richer blood supply than typically seen in pituitary adenomas. The patient declined radiation therapy. Consequently, our team performed EETS.

SFTs are recognized for their invasive growth, recurrence risk, and potential for metastasis. The prognosis largely depends on the extent of tumor resection. Metastasis rates can reach up to 70%, with common sites including the bone, lung, and liver (15, 16), Although the patient in our study showed no signs of metastasis, they underwent proton therapy post-surgery at the Shanghai Proton Heavy Ion Hospital. The treatment approach for this patient is detailed in our previous article (16).

This case also highlights the advantages of neuroendoscopy over traditional microscope surgery in transnasal procedures. Neuroendoscopy provides a more comprehensive field of view and offers significant benefits in terms of intraoperative hemostasis and thorough tumor removal.

(2) Primary Autoimmune Hypophysitis (PAH) is an autoimmune inflammation of the pituitary gland, distinct from secondary manifestations from other locations. The main histological subtypes of pituitary inflammation include LYH, granulomatous hypophysitis, xanthomatous hypophysitis, and IgG4 hypophysitis (17). LYH, a rare inflammatory lesion of the pituitary gland, is characterized by symptoms of hypopituitarism (18), and is difficult to diagnose. It mainly manifests as lymphocyte and plasma cell infiltration, with varying degrees of fibrosis in the pituitary gland and adjacent tissues (17).

Anatomically, LYH predominantly affects women, representing 69% of cases, especially during pregnancy or postpartum, with most cases noted in the final month of pregnancy or within the first 2 months post-delivery. The most frequently compromised hormone is adrenocorticotropic hormone (ACTH), followed by deficiencies in luteinizing hormone (LH), follicle-stimulating hormone (FSH), and thyroid-stimulating hormone (TSH). Growth hormone (GH) deficiency is relatively rare (19).

In clinical practice, LYH is often diagnosed based on clinical manifestations, biochemical/endocrine tests, and imaging examinations. These methods aid in identifying patients with LYH and guiding their treatment. However, when the diagnosis is uncertain and pathological findings may influence treatment decisions, a pituitary biopsy is recommended.

In Case 2, the patient, who was pregnant, had an MRI revealing a 30 mm × 22 mm solid-cystic lesion in the sellar region, extending through the diaphragm sellae and exhibiting a “snowman” sign (Figures 2A–D). The patient’s visual acuity had deteriorated significantly, prompting a transnasal neuroendoscopic resection of the pituitary lesion. During surgery, cystic fluid resembling that of a Rathke’s cyst was released. However, the surrounding tumor tissue showed pronounced fibrosis and a firm texture, markedly different from a typical pituitary tumor (Figure 2E). Immediate pathology indicated pituitary inflammation, but the specific subtype was not identified. To avoid permanent hypopituitarism after full mass removal, only a small sample of the lesion was excised for routine pathology. Histopathological examination confirmed the lesion as LYH (Figures 2J–M). Combining with literature reports (20–23), there is no relevant documentation on the combination of Hypophysitis and cyst. Unfortunately, we did not conduct pathological examination on the cystic fluid, and thus, cannot rule out whether it is a combination of Hypophysitis and Rathke’s cyst.

Due to limited knowledge about the disease and imaging findings that closely resemble those of a pituitary adenoma, accurately diagnosing LYH is challenging. Nonetheless, LYH can exhibit distinct imaging characteristics. MRI often reveals a notably enlarged pituitary gland with symmetrical uniform enhancement centered around the pituitary stalk. Considering the patient’s pregnancy status and the intraoperative findings of tumor tissue with pronounced fibrosis and a hard texture, a differential diagnosis becomes feasible. However, for most neoplastic masses, achieving a pre-operative diagnosis remains difficult. Enhanced sensitivity to such unusual masses is crucial, as it can influence surgical decisions and treatment approaches.

Following surgery, the patient underwent hormonotherapy. A three-month follow-up MRI suggested that the lesion had disappeared (Figures 2F–I). Currently, the patient’s hormone levels are normal. If the lesion had been entirely excised during surgery, it could have resulted in reduced pituitary hormones.

Spontaneous remission can occur in some LYH patients (18). If we encounter this scenario again, empirical hormone replacement therapy could be applied. Short-term treatment may significantly shrink the lesions in patients, leading to symptom alleviation. Therefore, preoperative diagnosis is critical, and more case studies are needed to gather sufficient diagnostic details.

(3) Cavernous Sinus Hemangiomas (CSHs) are rare benign vascular tumors, accounting for less than 2% of all cavernous sinus lesions (24, 25). In our case series, Case 3 demonstrated a sellar mass infiltrating the right cavernous sinus, partially encompassing the adjacent intracavernous internal carotid artery. The CT scan showed abnormal density in the pituitary fossa. MRI depicted a mass in the right parasellar region with T1WI iso-to-hypo signal, T2WI iso-to-hyper mixed signal, and DWI iso-signal. Although the tumor itself wasn’t significantly enhanced, its periphery was. The lesion measured 30 mm × 25 mm (Figures 3A–C).

The patient underwent EETS for treatment, use this surgical approach, we can clearly distinguish the relationship between the tumors in cavernous sinus and the pituitary gland and the internal carotid artery (Figure 3D). Relevant scholars reviewed the literature comparing EETS with frontotemporal craniotomy and stereotactic radiosurgery indicated that intrasellar-type CSHs could be safely removed by EETS without crossing the nerves in the cavernous sinus (5).

Post-surgery, the patient had no symptoms, normal eye movements, and hormone levels within the normal range. Postoperative MRI showed successful tumor removal (Figures 3E,F). Subsequent histopathological examination confirmed the lesion as CSH (Figures 3G,H).

In this case series, all three patients presented with conditions located near the pituitary fossa and closely associated with the pituitary gland. Differentiating these from pituitary tumors using imaging alone poses challenges. However, each case has distinct characteristics. Careful examination of preoperative imaging studies and clinical information can help minimize the rate of preoperative misdiagnosis.

1. Three cases of unusual lesions in the sphenoid sinus

Some tumors exhibit typical imaging characteristics, but their locations are highly unusual, as seen in Case 4 (OF), Case 5 (OF), and Case 6 (SSM).

(1) OF is a rare benign fibro-osseous neoplasm infrequently affecting the cranial vault or base. About 10% of OF cases occur in the maxilla, and it is rare for OF to involve the ethmoid sinuses and orbit (26, 27), The highest incidence is found in females aged 20–40 (27). However, in our case series, both patients we reported were male (Cases 4 and 5).

CT scans of OF patients typically show tumors swelling and expanding around the medullary cavity as a central focus (27). There is often a complete or incomplete bony casing along the tumor margin. Most patients present with masses that have well-defined boundaries, are typically singular, sometimes hemorrhaging, with some displaying focal mucinous degeneration. Certain lesions can be observed in the low signal area of T2WI, and secondary cystic changes vary with different capsule components, generally presenting with isointense T1WI signals and long T2WI signals (28). For instance, in Case 5, MRI showed low T1WI signal and variable signal on T2WI, which was significantly enhanced after enhancement (Figure 5). However, Case 4’s MRI presentation is not typical, with hyper-signal on T1WI (Figure 4B).

CT scans are crucial for this disease as they can reveal bone expansion and growth. MRI is sometimes superior in detecting soft tissue lesions, but it often fails to provide detailed information about bone changes.

OF is primarily differentiated from fibrous dysplasia, with the key distinctions being in their imaging characteristics and lesion borders. OF typically presents as an expansile growth centered around the medullary cavity, often involving a single bone with distinct borders. In contrast, fibrous dysplasia tends to exhibit a diffuse growth within the medullary cavity, frequently involving multiple bones, and the transition between the affected area and normal bone lacks clear demarcation (27, 29, 30). A definitive diagnosis is established through a combination of CT imaging and histopathological examination. When a single bone is affected, differentiating solely based on imaging can be challenging, making pathological confirmation necessary. The primary pathological characteristic of OF is the presence of small bony trabeculae (31).

During surgery for both patients, the sphenoid sinus tumors were decompressed for removal. Subsequently, the bone shell on the skull base or sphenoid bone was gradually peeled off, utilizing a diamond drill toward the tumor shell until the bone cortex was smoothened. Compared to traditional microscopic surgery, the advantages of EETS include direct visualization, enhanced visibility resulting in decreased intraoperative and postoperative morbidity (27).

Due to its location, the tumor benefits from a rich blood supply, presenting a heightened risk of intraoperative bleeding and complicating precise intraoperative tumor boundary determination. There were significant differences in preoperative MRI between the two patients, and cystic wall necrosis was present in Case 5 (Figures 5B–D), the blood supply of the two patients was significantly different, with the blood supply of the patient in Case 5 being more abundant than in Case 4.

Postoperative CT and MRI for Case 4 indicated satisfactory tumor resection (Figures 3D,E), while Case 5 revealed minimal residue in the sphenoid bone (Figure 5F). However, symptoms for both patients had fully resolved, and further follow-ups were planned. Postoperative pathological diagnosis confirmed OF.

(2) Mucoceles are benign, encapsulated, expansible, and locally invasive masses located within a paranasal sinus. They result from an accumulation of secretions and debris in the sinus cavity due to the obstruction of the sinus ostium and are filled with mucus and lined by epithelium. Sphenoid sinus mucoceles are relatively rare, accounting for only about 1% of all paranasal sinus mucoceles (32, 33).

Mucoceles are typically asymptomatic and are often discovered incidentally. However, due to compression of surrounding structures, symptoms like headache, deep orbital pain, and visual disturbance may occur (34), with headache being a common symptom in patients with a sphenoid mucocele.

The primary complaints in Case 6 were dizziness and discomfort in the left eye. The CT scan showed a lesion in the sphenoid sinus, notable dilation of the sphenoid sinus, and bone destruction on the left side, measuring 35 mm × 30 mm, compressing the left orbital wall. The MRI indicated a hyper-signal on T1WI, hypo-signal on T2WI, and enhancement on T1WI (Figures 6A–D). MRI is crucial in detecting mucoceles.

The treatment goal for a mucocele is to create a large ostium that allows drainage into the sphenoethmoidal recess. Transnasal endoscopic surgical resection was the chosen treatment method. This approach keeps the cavity open, preserving the continuity of the sinus mucosa with the nasal cavities, thereby reducing the risk of recurrence. The postoperative pathological diagnosis confirmed the presence of a mucocele (Figure 6F).

1. Two cases of PA preoperative MRI were misdiagnosed as Rathke’s cyst

PA are rare, comprising less than 1% of pituitary lesions, characterized by an infected purulent collection within the sellar turcica (35). In our case series, two cases of PA were inaccurately diagnosed as Rathke’s cyst through imaging alone.

MRI findings of a pituitary abscess typically present as a cystic lesion in the sellar region with enhanced cyst wall. Other cystic lesions of the pituitary, such as Rathke’s cleft cyst, cystic craniopharyngioma, or cystic pituitary adenoma, may also show ring enhancement around the lesion on MRI. Clinical presentation, however, aids in differentiation. Distinguishing these conditions can be challenging in the absence of clinical fever symptoms (36, 37).

Case 7 involved a 67-year-old woman with a one-month history of headache and intermittent fever. Her intermittent fever and cerebrospinal fluid examination suggested meningoencephalitis. In contrast, Case 8 involved a 72-year-old male patient who primarily complained of headaches and eye strain but did not present with a fever. Initially misdiagnosed as a cyst, the surgery revealed that the abscess in the internal region was connected with the sphenoid sinus mucosal abscess due to destruction of the sellar bottom bone. This discovery led to the revised diagnosis of pituitary abscess. Reviewing the preoperative MRI, we observed uniform enhancement at the edge of the saddle area, but DWI only showed localized limited diffusion with a high signal, unlike a typical brain parenchymal abscess, where the high signal in DWI is presented in clumps.

During surgeries, both patients had significant abscess discharge, and the pituitary fossa was repeatedly rinsed with hydrogen peroxide, and the bottom of the saddle was not repaired. Post-surgery no cerebrospinal fluid rhinorrhea observed and recovered quickly. Pathological smears showed abundant neutrophils and degenerated pituitary cells, but no bacteria were found in the microbial culture.

## Conclusion

In our case series, we observed that most lesions in the sellar region predominantly presented symptoms related to their occupying and pressing effects. Only one case, LYH, exhibited hypopituitarism and visual field defects. All patients described in this study underwent EETS, with no cases of cerebrospinal fluid leakage or postoperative complications, such as aggravated pituitary dysfunction and visual impairment. The advancements in endoscopic surgery demonstrate that EETS is a favorable option for sellar lesion surgery.

Unusual diseases in the sellar region often lead to preoperative misdiagnoses. In our case series, we shared the clinical manifestations, hormone levels, imaging features, surgical treatments, and patient prognoses of eight unusual cases in the sellar region. This information may assist researchers in improving preoperative diagnostic capabilities and in forming more effective treatment strategies for unusual cases.

## Data availability statement

The original contributions presented in the study are included in the article/supplementary material, further inquiries can be directed to the corresponding author.

## Ethics statement

The studies involving humans were approved by Ethical Committee of the Affiliated Suzhou Hospital of Nanjing Medical University. The studies were conducted in accordance with the local legislation and institutional requirements. The participants provided their written informed consent to participate in this study. Written informed consent was obtained from the individual (s) for the publication of any potentially identifiable images or data included in this article.

## Author contributions

JW: Writing – original draft. PD: Writing – original draft. JQ: Writing – original draft. YL: Writing – original draft. ZD: Writing – original draft. XH: Writing – original draft. YG: Writing – original draft. XT: Writing – review & editing. MM: Writing – review & editing.
